# Exercise through a cardiac rehabilitation program attenuates oxidative stress in patients submitted to coronary artery bypass grafting^*^

**DOI:** 10.1080/13510002.2017.1418191

**Published:** 2017-12-27

**Authors:** José Francisco Taty Zau, Rodrigo Costa Zeferino, Nádia Sandrine Mota, Gerez Fernandes Martins, Salvador Manoel Serra, Therezil Bonates da Cunha, Daniel Medeiros Lima, Basilio de Bragança Pereira, Emília Matos do Nascimento, Danilo Wilhelm Filho, Rozangela Curi Pedrosa, Roberto Coury Pedrosa

**Affiliations:** a Cardiology Department, University Hospital Clementino Fraga Filho, Rio de Janeiro, Brazil; b Cardiology Institute Edson Saad, Universidade Federal do Rio de janeiro-UFRJ, Rio de Janeiro, Brazil; c Biochemistry Department, Laboratory of Experimental Biochemistry, Universidade Federal de Santa Catarina-UFSC, Florianópolis, Brazil; d Instituto Aloysio de Castro-IECAC, Universidade Estadual do Rio de Janeiro-UERJ, Rio de Janeiro, Brazil; e Department of Biostatistics and Applied Statistics, Faculty of Medicine, Alberto Luiz Coimbra Institute of Graduate Studies and Research in Engineering, Universidade Federal do Rio de Janeiro-UFRJ, Rio de Janeiro, Brazil; f Ecology and Zoology Department, Universidade Federal de Santa Catarina-UFSC, Florianópolis, Brazil

**Keywords:** Coronary artery disease, myocardial revascularization, cardiac rehabilitation program, oxidative stress, antioxidants defenses

## Abstract

**Background:** Cardiovascular disease is the main cause of morbidity and mortality in the world and oxidative stress has been implicated in the pathogenesis. Cardiac rehabilitation in patients with coronary artery disease submitted to coronary artery bypass grafting may prevent cardiovascular events probably through the attenuation of oxidative stress. The aim of this study was to evaluate the benefits of a cardiac rehabilitation program in the control of the systemic oxidative stress.

**Methods:** The studied population consisted of 40 patients, with chronic stable coronary artery disease submitted to coronary artery bypass grafting, who attended a cardiac rehabilitation program. Biomarkers of oxidative stress were evaluated in the blood of these patients at different moments.

**Results:** After the onset of cardiac rehabilitation, there was a significant and progressive decrease in thiobarbituric acid reactive substances levels and protein carbonyls, an initial increase and subsequent decrease in superoxide dismutase, catalase and glutathione peroxidase activities. Also, a progressive increase of uric acid, while ferric reducing antioxidant power levels increased only at the end of the cardiac rehabilitation and a tendency to increase of glutathione contents.

**Conclusions:** The results suggest that regular exercise through a cardiac rehabilitation program can attenuate oxidative stress in chronic coronary artery disease patients submitted to coronary artery bypass grafting.

## GRAPHICAL ABSTRACT

**Figure:**
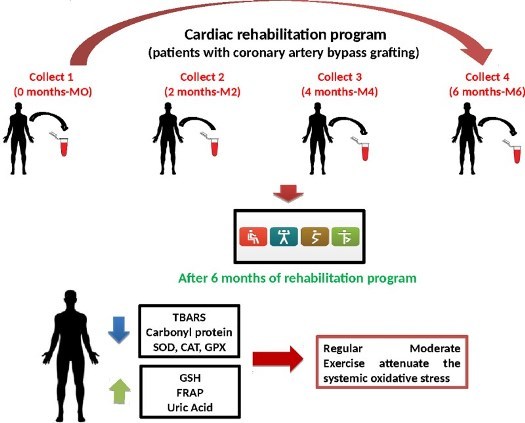

## 1. Introduction

Cardiovascular disease is the main cause of mortality, morbidity and disability in the world [1]. Atherosclerosis, the most common pathological process associated with cardiovascular disease, has a high state of oxidative stress characterized by lipid and protein oxidation [2,3]. The oxidation of low-density lipoprotein is the first step in the development and progression of atherosclerosis and coronary artery disease [2,4].

Oxidative stress is defined as the imbalance between oxidants and antioxidants in favor of the former, characterized by the increase of the production of reactive oxygen species (ROS), while being frequently also characterized by a depletion of antioxidants produced by drug treatments [5–7]. Oxidative stress can cause damage to important biomolecules, such as DNA, lipids and proteins [6]. Although much described in the literature, there still remains a certain debate on oxidative stress and exercise, since exercise promotes increased ROS production causing oxidative stress with implications in the pathogenesis of cardiovascular disease [8–10]. On the other hand, a regular and moderate exercise promotes a favorable adaptation to the organism by balancing the production of antioxidants thereby compensating the oxidative stress [11–13]. Therefore, this study aimed to evaluate the eventual benefits of a cardiac rehabilitation program in the control of the systemic oxidative stress.

## 2. Methods

The present study is Quasi-experimental with time series [14]. The study population consisted of patients with chronic stable coronary artery disease submitted to coronary artery bypass grafting, beginning in a cardiac rehabilitation program between 6 months and 1 year after discharge. The study period was from January 2014 to January 2016. We included patients with a history of coronary artery bypass grafting, indicated to begin cardiac rehabilitation program between 6 months and 1 year after discharge, those with two or more bridges (saphenous/breast), ejection fraction equal to or greater than 35%, and patients with food and medication defined as standard that were not in use of vitamin C, vitamin E or other dietary supplements containing antioxidants. Excluded patients were those under 18 years, pregnant women, smokers, those with initial admission protocol incomplete, with clinical or laboratory findings suggestive of acute renal disease or chronic liver disease, refractory heart failure or thyroid dysfunction, at any stage with unstable medical conditions and those with severe orthopedic or neurological problems.

### 2.1. Cardiac rehabilitation program

The cardiac rehabilitation program lasted 6 months, with a total of 50 exercise sessions twice a week, with a daily period of 60–75 min, divided as follows: 5 min of heating, followed by 20 and 10 min of aerobic exercise on a treadmill and bike, respectively, 20 min of weight training, and finally 5 min of stretching. The sessions encompassed dynamic exercises performed on treadmill and cycle ergometer, strength exercises in specific strength training equipment, balance and flexibility exercises. The time interval from the last meal (dinner) and the beginning of the physical exercise, i.e. before the blood collection, was approximately 12 h.

The exercise intensity in the first session was based on the functional capacity determined by the number of METs (Unit equivalent to consumption of 3.5 ml/oxygen/kg/min) assessed in the stress test and the heart rate range set for training. Increases or decreases in subsequent exercise intensity were due to heart rate, Borg scale (0–10) and exercise tolerance achieved by the patient. The intensity was increased from identification of subjective reducing feeling of tiredness (Borg scale 0–10) to the same intensity of exercise. During aerobic exercise, the patients were instructed to exercise within the target zone of predetermined heart rate. To determine the intensity of heart rate training, the Karvonen adapted equation was used, with the corresponding lower limit to 60% of heart rate reserve and the upper limit to 80% heart rate reserve.

The samples for analysis of the biochemical markers of oxidative stress were measured in the following moments (M): before the beginning of the rehabilitation program (M0), at the second month after the beginning (M2), at the fourth month (M4) and at the sixth month (M6), immediately after the rehabilitation program. During the study, lipid damage marker (TBARS: Thiobarbituric Acid Reactive Substances), protein damage marker (PC: Protein Carbonyl) and antioxidants, such as the enzymatic activity of SOD (Superoxide Dismutase), CAT (Catalase) and GPx (Glutathione peroxidase), as well as non-enzymatic antioxidants, such as the levels of reduced glutathione (GSH), uric acid and FRAP (ferric reducing antioxidant power) were evaluated. Blood samples (10 ml) from all patients were collected. At baseline, patients were stratified by coronary artery bypass time (6–8 months, 8–10 months and 10–12 months), in order to check if myocardial revascularization interferes with the results in relation to oxidative stress markers.

### 2.2. Preparation of samples

Blood samples were collected always during the morning, intravenously under vacuum after about 12 h of fasting and 72 h after the last exercise session, using two tubes containing EDTA for plasma and a tube for serum from the antecubital fossa. The first tube containing EDTA withdrew 200 µl of whole blood and that was mixed with 800 µl of trichloroacetic acid 12% (w/v) (dilution 1:5) by cryotube alfalab/2 ml for measurement of GSH; another tube retreated 400 µl of whole blood that was mixed with 1600 µl of distilled water (dilution 1:5) and placed in cryotube alfalab/2 ml for measurements of SOD, CAT and GPx. The second tube with EDTA was centrifuged at 5000 *g* for 10 min to separate the plasma, and subsequently added to the plasma cryovial alfalab/2 ml for measuring the levels of TBARS and PC. The serum tube was also centrifuged at 5000 *g* for 10 min to separate serum and then placed in cryotube alfalab/2 ml for measuring uric acid levels. After this process of preparation of the samples, they were shipped (Rio de Janeiro to Florianopolis) in Styrofoam with dry ice at temperatures between −8° and −2° and then stored in a freezer at −80° until were analyzed.

### 2.3. Markers of oxidative damage

Lipid peroxidation was assessed by the measurement of substances that react with TBA using the thiobarbituric acid (TBARS) method, as described Ohkawa et al. [15]. Oxidative damage to proteins (PC) was quantiﬁed as PC at 360 nm as originally described by Levine et al. [16]. In this assay, carbonyl groups from proteins in the sample react covalently with 2,4-dinitrophenylhydrazine in acid, which leads to the formation of a 2,4-dinitrophenylhydrazone product.

### 2.4. Determination of antioxidant enzyme activity

SOD activity was measured spectrophotometrically at 480 nm according to the method of Misra and Fridovich [17], modified by Boveris et al. [18] by the oxidation of adrenaline (change of pH 2.0 to pH 10.0) forming superoxide anion and a pink chromophore, the adrenochrome, where the enzyme present in the sample retards its formation. CAT activity was determined according to the method described by Aebi [19], which measures the rate of decomposition of hydrogen peroxide, at 240 nm for 20 s by the enzyme present in the sample. The determination of GPx activity was performed according to the method of Flohé and Gunzler [20], where the reaction is based on the reduction of terc-butyl hydroperoxide by the oxidation of GSH and formation of GSSG catalyzed by GPx.

### 2.5. Determination of non-enzymatic antioxidant defenses

The reduced GSH content in whole blood was evaluated by the method of Beutler et al. [21] determining the non-protein thiols, since GSH is around 95% of these thiols. The content of serum uric acid was measured by commercial kit Analisa^®^, in which uric acid is oxidized by uricase to allantoin, CO_2_ and H_2_O_2_. Through an oxidation reaction catalyzed by peroxidase, H_2_O_2_ reacts with the formed dicloro hidroxi benzeno sulfonate and 4-aminoantipyrine, producing are compound antipirilquinonimina. The antioxidant capacity of plasma (FRAP) was determined according to Benzie and Strain [22,23], which measures the ability of plasma to reduce the Fe^+++^ to Fe^++^ in a redox reaction coupled to a colorimetric method.

## 3. Ethical considerations and statistical analysis

The study was approved by the Research Ethics Committee of the State Institute of Cardiology Aloysio de Castro (CEP-IECAC), under the protocol number 20554913.0.0000.5265, and conforms to standards currently applied by the Brazilian National Committee for Research Ethics and to the ethical guidelines of the 1975, *Declaration of Helsinki*. A written informed consent was obtained from all subjects included in this study following Good Clinical Practices.

Data were expressed as mean and standard deviation (mean ± SEM). The difference between the moments (M0>>>M2/M4/M6) of TBARS, PC, SOD, GPx, GSH and uric acid were tested by analysis of variance with repeated measures one entry (one-way ANOVA) and Holm–Sidak, after testing the assumptions of normality and sphericity by the D’Agostino and Pearson test. The data from the CAT and FRAP experiments, which did not show a normal distribution were analyzed by Friedman test and Dunn’s of multiple comparisons. The minimal level of significance was set at 5% and the statistical analysis was performed using the Graph Pad Prism software 6 (Graph pad Inc., San Diego, CA, USA).

## 4. Results

All patients of the present study showed chronic stable coronary disease, were sedentary, aged between 42 and 75 years and 29 were male. About 52% were overweight, 68% with comorbidities, more than 70% of patients were using converting enzyme angiotensin inhibitors, statins and β-blockers. The average left ventricular ejection fraction was 38%. No patient had thyroid dysfunction or anemia and concomitant infections (Table 1). During the study, all the patients kept the same medication.

**Table 1.** General characteristics of the population.

Variable	Patients
Age (average)	61.1 (42–75)
Male (%)	72.5
BMI (kg/m^2^)	25.4 (23.1–28.3)
Comorbidities (HAS/DM/DLP) (%)	67.5
EF (%)	38 ± 3
ACE inhibitor (%)	72.5
β-blocker (%)	95.0
CCB (%)	2.5
Diuretic (%)	12.5
Statin (%)	82.5
Sedentary (%)	100
Hemoglobin (mg/dl)	11.9–14.2
Leukocytes (cells/mm^3^)	5300 ± 800
STH (mU/l)	2.3–3.1

Note: BMI: body mass index; HBP: high blood pressure; DM: diabetes mellitus; DLP: dyslipidemia; EF: ejection fraction; TSH: thyroid stimulating hormone; CCB: calcium channel blocker; ACE: angiotensin converting enzyme inhibitor.

Values are expressed as mean ± SEM, *n* = 40.

In assessing the behavior of oxidative damage markers (TBARS and PC levels) and antioxidant defenses (GSH, SOD, GPx, Uric acid and FRAP) in different times after hospital discharge and before cardiac rehabilitation program (6–8 months, 8–10 months, 10–12 months), no statistical difference between them was found.

**Figure 1.**
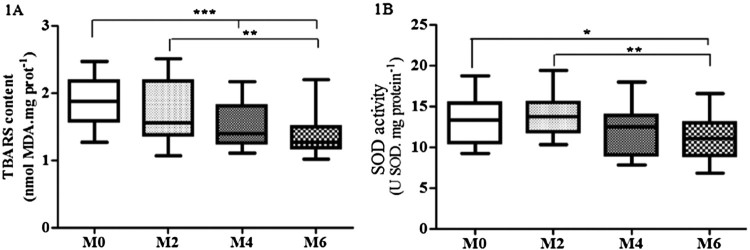
Levels of lipid peroxidation (TBARS) (A) and SOD activity (B) in plasma along the cardiac rehabilitation: M0 (assessment before cardiac rehabilitation; *n* = 40); M2 (after 2 months: *n* = 35); M4 (after 4 months: *n* = 30); M6 (after 6 months: *n* = 28). The one entry (one-way ANOVA) and Holm–Sidak, after testing the assumptions of normality and sphericity by the D’Agostino and Pearson test was performed for analysis of the four moments, followed by the Dunn’s test and the statistical difference was denoted by *p < 0.05, **p < 0.01 and ***p < 0.001.

However, statistical analysis demonstrated by ANOVA followed by the Holm–Sidak’s test showed a significant and progressive decrease in the levels of TBARS after cardiac rehabilitation at all intervals examined: M0–M2 (↓21%; *p* = ns); M0–M4 (↓56%; *p* < 0.001) and M0–M6 (↓74%; *p* < 0.001) (Figure 1(A)). The ANOVA test also revealed no significant differences in PC levels after cardiac rehabilitation at all moments, despite a not significant downward trend among M2/M4/M6 (Table 2).

**Table 2:** . Markers of oxidative stress and activity of antioxidants enzymes measured in four moments during cardiac rehabilitation program: M0 (assessment before cardiac rehabilitation; *n* = 40); M2 (after 2 months: *n* = 35); M4 (after 4 months: *n* = 30); M6 (after 6 months: *n* = 28).

	M0	M2	M4	M6
TBARS (nmol MDA mg protein^-1^)	2.50 ± 0.34	2.35 ± 0.55	2.00 ± 0.40	1.97 ± 0.51
CP (µmol mg^-1 ^protein^–1^)	1.36 ± 0.28	1.33 ± 0.23	1.21 ± 0.20	1.24 ± 0.27
SOD (U ml^-1^)	13.41 ± 2.85	14.00 ± 2.43	12.18 ± 2.89	11.13 ± 2.78
CAT (mmol min^-1^ mg protein^-1^)	0.32 ± 0.08	0.35 ± 0.09	0.31 ± 0.11	0.26 ± 0.10
GPx (mmol min^-1^ mg protein^-1^)	0.05 ± 0.01	0.06 ± 0.02	0.05 ± 0.01	0.04 ± 0.01
GSH (pmol ml^-1^ mg protein^-1^)	0.54 ± 0.22	0.55 ± 0.31	0.58 ± 0.33	0.68 ± 0.36*
Uric acid (mg dl^-1^)	4.11 ± 0.67	4.30 ± 0.57	4.30 ± 0.58	5.14 ± 0.75
FRAP (µmol l^-1^)	4.32 ± 2.87	4.06 ± 1.91	5.1 ± 4.17	23.17 ± 19.53

Note: TBARS: thiobarbituric acid reactive substances; CP: carbonyl proteins; GSH: reduced glutathione; CAT: catalase; GPx: glutathione peroxidase; SOD: superoxide dismutase; FRAP: ferric reducing antioxidant power; Values are expressed as mean ± SEM. M0 = Initial moment; M2, M4 and M6: after 2, 4 and 6 months of the cardiac rehabilitation program.

All values are expressed as mean ± SEM.

Significant difference in SOD activity at all moments was observed, only a not significant initial increase in M2 and a subsequent decrease at the end of cardiac rehabilitation program, showed by ANOVA followed by Holm–Sidak’s test (Figure 1(B)). CAT showed decreased levels after cardiac rehabilitation from M4; after an initial increase in M2, without significance demonstrated by Dunn’s test, after the Friedman test (Table 2). ANOVA also revealed no significant difference in GPx enzyme activity after cardiac rehabilitation at all moments, only a slight decrease from M0 to M4/M6, after a small increase in M2 (Table 2).

**Figure 2.**
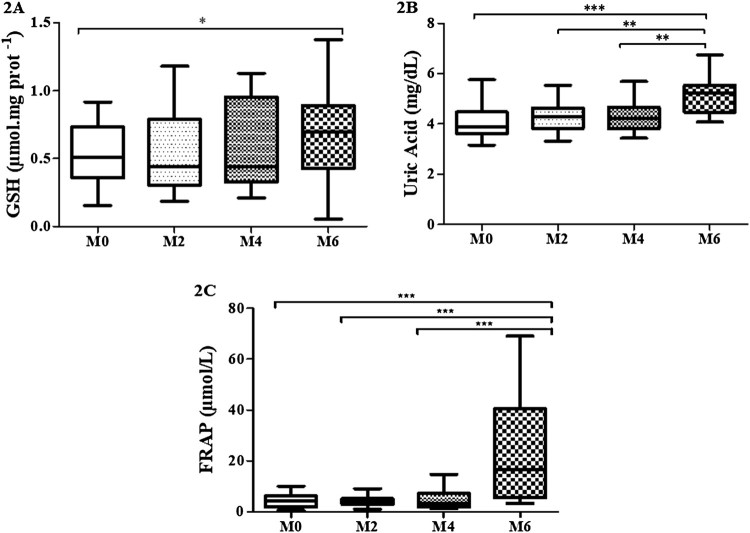
Levels of GSH (A), acid uric (B) and FRAP (C) in whole blood along cardiac rehabilitation: M0 (assessment before cardiac rehabilitation; *n* = 40); M2 (after 2 months: *n* = 35); M4 (after 4 months: *n* = 30); M6 (after 6 months: *n* = 28).The one entry (one-way ANOVA) and Holm–Sidak, after testing the assumptions of normality and sphericity by the D’Agostino and Pearson test was performed for analysis of the four moments, followed by the Dunn’s test and the corresponding statistical differences. For FRAP data, the Friedman test was performed for analysis of the four moments, followed by the Dunn’s test and the statistical difference was denoted by *p < 0.05, **p < 0.01 and ***p < 0.001.

The ANOVA test also revealed a significant difference in the levels of whole blood GSH after cardiac rehabilitation from M0 to M6 (↑25%; *p* < 0.01), which was confirmed by the Holm–Sidak’s test (Table 2) (Figure 2(A)). Statistical analysis showed a progressive and significant increase in uric acid levels after cardiac rehabilitation at all moments, especially from M0 to M6 (↑87%; *p* < 0.001), by ANOVA followed by Holm–Sidak’s test (Table 2) (Figure 2(B)). Moreover, the cardiac rehabilitation promoted a remarkable significant increase (*ca*. fivefold) in the levels of FRAP, at the end of the study (M6) compared to the other previous moments (↑536%; *p* < 0.001), revealed by the Friedman test followed by Dunn’s test (Table 2) (Figure 2(C)).

## 5. Discussion

The oxidative marker of lipid damage (TBARS), which was high at baseline (M0), progressively decreased after 2 months of cardiac rehabilitation (M2; ↓21%), stabilizing after 4 and 6 months (M4 and M6) compared to the initial moment (M0), indicating attenuation of the systemic oxidative stress influenced by regular physical exercise. Accordingly, Melek et al. [24] found that oxidative stress (TBARS levels) increased during coronary artery bypass grafting with extracorporeal circulation, while Gwzdzinski et al. [25] evaluating surgical revascularized patients previously to the cardiac rehabilitation also observed increased TBARS levels, while after the cardiac rehabilitation a decrease in such values was found, thereby corroborating our finding.

The other oxidative damage marker PC showed a similar and consistent profile of lipid peroxidation, although did not reach significant differences, probably related to the relatively low number of samples together with the high dispersion (variance) found in the values obtained for this parameter, despite they were carried in triplicate. Levine, based on the original assay for PC levels [16], established that the increase of its levels is associated with increased oxidative stress. Pinho and collaborators evaluating the effect of regular exercise on oxidative stress in rats submitted to acute exposure to coal combustion followed by 12 weeks of exercise, observed a decrease in PC well as in lipid peroxidation levels [26]. Accordingly, as mentioned above, a tendency to decrease was also observed in this marker of oxidative damage to proteins in our study corresponding to each period of evaluation after the onset of cardiac rehabilitation.

GSH levels in whole blood were stable at M2 compared to the time zero (M0), while showed an upward trend from 4 months of rehabilitation (M4). Even considering the lack of significance of these results, is possible to infer that the rehabilitation could promote a slight stability at the final moment (M6) of monitoring of this important non-enzymatic endogenous antioxidant, after its initial likely depletion measured at 2 months (M2). It is interesting to note that GSH is essential for the function of GPx [27], and that the activity of this enzyme did not show significant differences during all the analyzed moments, even if GSH levels were stable 2 and 4 months after the onset of the cardiac rehabilitation.

Consistent with this GSH profile, a similar response was also displayed at M6 for the analysis of plasma total antioxidant capacity (FRAP) and uric acid along the cardiac rehabilitation, thus corroborating the results obtained for GSH. In particular, it is important to note that FRAP and uric acid precisely assess this antioxidant capability restricted to non-enzymatic endogenous antioxidants, which includes GSH and several other exogenous or nutritional antioxidants, such as the antioxidant vitamins C, E, besides polyphenols, some hormones and others [28,29].

The three antioxidant enzymes here examined, which are considered the main triad in the detoxification of ROS in all organisms that use oxygen as oxidant [28], showed a similar profile and consistent with the development of cardiac rehabilitation by the patients. Comparatively to M0, SOD, CAT and GPx activity showed a small increase after 2 months (M2) conducting exercises, starting to decline after 4 months (M4) and all showed relatively lower values at 6 months (M6). The initial increase detected in these antioxidant enzymes is supported in fact that the organism after beginning an exercise program get around 8–12 months to accomplish favorable adjustments [12,13,30]. In other words, the response of the antioxidant enzymes examined in our study indicates, even though revealing no significance at all the moments examined, that the cardiac rehabilitation is promoting a progressive restoration of the antioxidant systemic capacity of the patients [28]. Consequently, after 6 months of monitoring cardiac rehabilitation, it was possible to see a stabilization of enzyme induction below the initial levels. Such regulation of the antioxidant enzymes was probably due to the decline of oxidative damage markers associated with the recovery of non-enzymatic endogenous antioxidants [27,31] GSH and uric acid, which were corroborated by the evaluation of FRAP levels, therefore attenuating the systemic oxidative stress of these patients.

Comparing the oxidative stress markers at moments prior to cardiac rehabilitation, no statistical differences were found among them, so that allow us to infer that probably the time from the surgery to cardiac rehabilitation did not interfere in the results of this study. As confirmed by other authors, it is known that during coronary artery bypass grafting, there is an increased systemic inflammatory response and a consequent imbalance between oxidants and antioxidants due to the so-called ischemia and reperfusion injury, which is subsequently controlled by the organism response after reperfusion [24], and assisted by the action of drugs such as β-blockers [32,33], statins and angiotensin converting enzyme inhibitors [34].

Regular exercise can improve total antioxidant capacity by modulating the synthesis of enzymatic antioxidants as those examined in the present study, as well as non-enzymatic antioxidant, while decreasing markers of oxidative damage such as lipid peroxidation, thereby also decreasing the systemic oxidative stress [13]. Such antioxidant response is corroborated by our results and is shared by other related studies, which highlight the positive influence of regular exercise on an overall improvement of the antioxidant capacity, especially after consecutive sessions of exercise, consequently promoting a progressive decline of oxidative stress [12,13,30].

Limitation

Drugs as β-blockers [32], Statins [33] and of Angiotensin converting enzyme inhibitors [34] could interfere positively in oxidative stress, although we believe that they did not interfere in the results of the present study, since the patients were already taking the medication at least for 6 months before joining the cardiac rehabilitation program.

We believe that the loss of the patients during the present study should not influenced the results, since most of the measurements showed a fairly similar profile, irrespective of the number of the patients.

## 6. Conclusion

In summary, the oxidative damage markers revealed a consistent decrease concomitant to a progressive increase in the non-enzymatic antioxidants examined in the present study. Therefore, it is possible to infer that regular moderate exercise through a cardiac rehabilitation program is able to attenuate the systemic oxidative stress in chronic coronary artery disease patients submitted to coronary artery bypass grafting, while preventing further oxidative damage through a general improvement in endogenous antioxidants. The main finding obtained in this study was the attenuation on lipid damage markers (TBARS levels), combined with a tendency on the attenuation of protein damage (PC levels), as well as increases in GSH and urate levels together with a marked increase in plasma total antioxidant capacity (FRAP levels) after 6 months of the onset of the rehabilitation program.
